# Immunocytochemical Characterization of Alzheimer Disease Hallmarks in APP/PS1 Transgenic Mice Treated with a New Anti-Amyloid-****β**** Vaccine

**DOI:** 10.1155/2013/709145

**Published:** 2013-09-09

**Authors:** Iván Carrera, Ignacio Etcheverría, Yi Li, Lucía Fernández-Novoa, Valter Lombardi, Carmen Vigo, Hector H. Palacios, Valery V. Benberin, Ramón Cacabelos, Gjumrakch Aliev

**Affiliations:** ^1^Department of Neurosciences, EuroEspes Biotechnology, 15165 La Coruna, Spain; ^2^Department of Human Sciences, Texas A&M University-Kingsville, Kingsville, TX, USA; ^3^Atlas Pharmaceuticals, Sunnyvale, CA, USA; ^4^National Institute on Aging, National Institutes of Health, Baltimore, MD, USA; ^5^Medical Center of the Administration of the President of the Republic of Kazakhstan, Astana, Kazakhstan; ^6^EuroEspes Biomedical Research Center, Institute for CNS Disorders and Genomic Medicine, 15165 La Coruna, Spain; ^7^“GALLY” International Biomedical Research Consulting LLC, San Antonio, TX, USA; ^8^Department of Health Science and Healthcare Administration, University of Atlanta, Atlanta, GA, USA

## Abstract

APP/PS1 double-transgenic mouse models of Alzheimer's disease (AD), which overexpress mutated forms of the gene for human amyloid precursor protein (APP) and presenilin 1 (PS1), have provided robust neuropathological hallmarks of AD-like pattern at early ages. This study characterizes immunocytochemical patterns of AD mouse brain as a model for human AD treated with the EB101 vaccine. In this novel vaccine, a new approach has been taken to circumvent past failures by judiciously selecting an adjuvant consisting of a physiological matrix embedded in liposomes, composed of naturally occurring phospholipids (phosphatidylcholine, phosphatidylglycerol, and cholesterol). Our findings showed that administration of amyloid-**β**_1−42_ (A**β**) and sphingosine-1-phosphate emulsified in liposome complex (EB101) to APP/PS1 mice before onset of A**β** deposition (7 weeks of age) and/or at an older age (35 weeks of age) is effective in halting the progression and clearing the AD-like neuropathological hallmarks. Passive immunization with EB101 did not activate inflammatory responses from the immune system and astrocytes. Consistent with a decreased inflammatory background, the basal immunological interaction between the T cells and the affected areas (hippocampus) in the brain of treated mice was notably reduced. These results demonstrate that immunization with EB101 vaccine prevents and attenuates AD neuropathology in this type of double-transgenic mice.

## 1. Introduction 

Alzheimer disease (AD) is the most common chronic neurodegenerative disorder, affecting almost one-third of elderly individuals in the Western countries [1]. AD clinical phenotype includes progressive memory loss, personality changes, language problems, spatiotemporal confusion, and a general decline in cognitive function, displaying characteristic brain pathological hallmarks characterized by accumulation of amyloid-*β* (A*β*) peptide (amyloid plaques), neurofibrillary tangles which are composed mainly of paired helical filaments with hyperphosphorylated tau proteins and neuronal and synaptic loss with preferential damage in neocortex, hippocampus, and entorhinal region [2]. Neuroinflammation, gliosis, and impaired regulation of oxidative stress and metabolic dysfunction are also present in secondary pathogenic events [3]. Over 100 different genes distributed in the human genome are potentially associated with AD [4–7]. In the past decade, numerous immunotherapeutical studies have demonstrated that some forms of A*β*-aggregated peptides play an important role in the pathogenesis of this neurodegenerative disease [8, 9]. These results led to experimental therapeutic immunization strategies to reduce cerebral A*β*, using AD mouse models. Among the therapeutical approaches, there exists the targeting of the enzymes that cleave APP to A*β* peptides in order to reduce their deposition or the inhibiting of their aggregation into insoluble deposits by clearance of A*β* peptides from the brain [10]. Transgenic mice expressing mutated forms of the gene for the human amyloid precursor protein (hAPP) and show a marked elevation in A*β*-protein level and A*β* deposition in the cerebral cortex and hippocampus [11–13] and develop similar neuropathological hallmarks to those observed in AD brains. Presenilin-1 (PS1) mutant transgenic mice display an increased A*β*_42_ peptide formation, potentiating amyloid deposition in Tg2576 APP mice at 6 months of age [14]. Therefore, double-transgenic mice derived from the coexpression of these mutated genes (APP/PS1) have demonstrated a markedly accelerated accumulation of A*β* deposits compared with single APP-transgenic mice [15–19]. Taking advantage of the potential aspects of this double-transgenic mouse line, numerous studies have used this particular AD mouse model to investigate emergent therapies to prevent and/or reduce the neuropathological features of AD. 

In the past few years, different therapeutical approaches have been performed to modulate the amyloid brain depositions in APP-transgenic mice, including restricted administration of pharmaceutical agents [20], rich cholesterol diet [21], caloric diet [22], and intensive exercise [23]; however, A*β*-based immunotherapy has been shown to be the most promising research field in reducing amyloid loads [24–28] and reversing memory deficits [9, 10, 29] in AD mouse models. Both active (A*β* peptides) and passive (A*β*-specific antibodies) immunizations have been reported to achieve a certain degree of efficiency in reducing brain A*β* deposits in AD mouse models [30]. Based on previous preclinical results, Elan and Wyeth initiated a clinical trial of active immunization with aggregated synthetic A*β*_1−42_ peptide/QS21 adjuvant against A*β* in patients with AD in 2001. This clinical trial was interrupted because of signs of meningoencephalitis in ~6% of immunized subjects [31], probably induced by an extensive T-cell-mediated immune response [32, 33]. Remarkably, patients with an abbreviated immunization protocol generated anti-A*β* antibodies, reducing cerebrospinal levels of tau, and reported a slower cognitive decline [34, 35]. All these data have been used in subsequent immunotherapeutic experiments. Because increasing evidence suggests that T-cell reactivity, A*β* burden levels, and cognitive function deficits are the main events that should be addressed to attenuate several hallmarks of AD mouse models, we developed a novel immunogen-adjuvant configuration with the potential to prevent and reduce A*β* deposits and avoid the massive activation of T-cell-mediated autoimmune response that may cause meningoencephalitis. In the present study, we analyze the neuropathological effects of a novel active immunization vaccine against amyloid plaques either before (prevention) or after (treatment) the onset of the AD hallmarks in mouse models. To examine both effects, APP/PS1 mice were inoculated with amyloid-*β* and sphingosine-1-phosphate emulsified in liposome complex and then studied by neuropathological markers. Our results indicate that the present vaccine halts the development and markedly reduces *β*-amyloid-plaque formation in (APP/PS1) double-transgenic mice and also attenuates the main inflammatory background present in affected areas of the brain.

## 2. Materials and Methods

### 2.1. Animals

Double-transgenic mice used in this study express a chimeric mouse/human amyloid precursor protein (Mo/HuAPP695swe) and a mutant human presenilin 1 (PS1-dE9) both directed to CNS neurons. These two constructs were coinjected into B6C3HF2 pronuclei, and insertion of the transgenes occurred at a single locus. Founder line 85 was obtained, and the resulting colonies were maintained as a hemizygote by crossing transgenic mice with B6C3F1/J mice, which were then purchased from The Jackson Laboratory. All mice were housed in plastic cages (2/3 mice/cage) with free access to food and water and were maintained under controlled conditions of humidity (50 ± 10%), light (12/12 hr light/dark cycle), and temperature (23°C ± 1). All experimental procedures were conformed to the guidelines established by the European Communities Council Directive of 24 November 1986 (86/609/EEC) and by the Spanish Royal Decree 1201/2005 for animal experimentation and were approved by the Ethical Committee of the EuroEspes Foundation. 

### 2.2. Experimental Design

**Figure 1:**
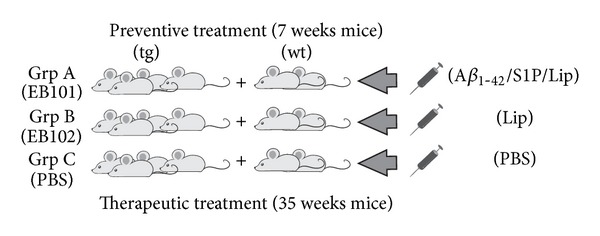
Diagram of the experimental protocol. EB101 vaccine was injected subcutaneously (s.c.) into mice of group A from 7 weeks of age (preventive treatment/phase I) or 35 weeks of age (therapeutic treatment/phase II), respectively, for 7 months. Mice of groups B and C were also injected s.c. with EB102 or PBS following the previous protocol.

In the present study, two experimental phases were performed, depending on the immunization treatment (Figure 1): a preventive immunization of mice at 7 weeks of age (before the onset of AD-type neuropathologies; phase I) and a therapeutic immunization of mice at 35 weeks of age (when neuropathological hallmarks were well established, phase II). Mice were randomly divided into these two experimental phases, each one with the following three treatment groups: phase I: group A consisting of 8 mice (6 transgenic mice and 2 wild-type mice) that were immunized with a cocktail of synthetic human A*β*_1−42_/S1P/liposome (EB101); group B consisting of 8 mice (6 transgenic mice and 2 wild-type mice) that were immunized with liposome alone (EB102); and group C consisting of 6 mice (4 transgenic mice and 2 wild-type mice) that were inoculated with PBS (control). Phase II: groups A, B, and C, consisting of the same number of mice as phase I, were treated with the same immunization protocol. Mice were immunized with nine injections for seven months and then kept for 2 additional months before sacrifice. Mice were 11 months old at the end of the preventive treatment and 18 months old at the end of the therapeutic treatment. 

### 2.3. Experimental Procedure

In the preparation of A*β*_1−42_, two milligrams of A*β*_1−42_ human (TOCRIS bioscience; Tocris Cookson Ltd.; this A*β*_1−42_ corresponds to the human form of the predominant amyloid beta-peptide found in the brains of patients with AD) were dissolved in 0.9 mL water and made up to 1 mL by adding 0.1 mL of 10x PBS. 

### 2.4. Preparation of Liposomes

Liposomes were prepared from 1,2-Dioleoyl-sn-Glycero-3-Phosphocholine (DOPC), 1-Palmitoyl-2-oleoyl-sn-glycero-3-phosphatidylglycerol, Sodium Salt (POPG), and cholesterol (CH; Northern lipids INC.), plus/minus D-erythro-sphingosine-1-phosphate (S1P), (AVANTI at 0.3/0.3/0.39/0.01, molar ratio, resp.). One hundred milligrams of each lipid were dissolved in 1 mL of chloroform and stored at −20°C until the preparation of the S1P liposomes; 10 mg was dissolved in 1.5 mL (2 : 1 chloroform/methanol) and stored at −20°C until use. Lipids, in the final proportion indicated above, were thoroughly mixed in the organic solvent, evaporated under nitrogen. The corresponding mixture containing total lipid 100 mg and 0.6 mg of S1P was resuspended in autoclaved ultrapure water and thoroughly vortex-mixed until a milky solution was formed or the so-called “multilamellar vesicles” (MLV). Small (SLV) or single unilamellar vesicles (SUV) were subsequently prepared by sonicating the MLV for 2 minutes at 30-second intervals in an ice bath until the solution was clear and centrifuging at 2500 g/15 minutes to eliminate debris. The SUV preparation was freeze-dried and resuspended in PBS-containing A*β* (10 mg) and thoroughly mixed. The freeze-dried mixture was resuspended in the corresponding amount of autoclaved ultrapure water, ready for immunization. 100 *μ*L of EB101 was used for the immunization step. Liposomes containing S1P and A*β* are referred to as EB101 and without S1P and A*β* are referred to as EB102.

### 2.5. Preparation of EB101 Liposomal Formulation

We combined the use of a biologically active lipid, sphingosine-1-phosphate (S1P), with amyloid beta-peptide, and we also changed the adjuvant previously used for a liposomal one that had been successfully used for other vaccines, including influenza. This new liposomal vehicle acts as a matrix to solubilize and deliver amyloid beta-peptide and S1P as adjuvant.

### 2.6. Preparation of Empty Liposomes (EB102)

The same steps as for EB101 preparation were followed to prepare EB102. This liposomal mixture contains 1,2-Dioleoyl-sn-Glycero-3-Phosphocholine (DOPC), 1-Palmitoyl-2-oleoyl-sn-glycero-3-phosphatidylglycerol, Sodium Salt (POPG), and cholesterol (CH) in the same concentrations as EB101, but it does not contain S1P or amyloid beta-peptide. 

### 2.7. Immunization Procedures

APP/PS1 tg mice were inoculated intraperitoneally with 100 *μ*L per injection of amyloid-*β* and sphingosine-1-phosphate emulsified in liposome complex (group A), liposome complex alone (group B), or PBS (group C), during seven months (9 injections). 

### 2.8. Immunohistochemistry

While anesthetized, the animals were perfused transcardially, first with NaCl solution and then with 4% paraformaldehyde, and their brains were excised and immersed in the same fixative for 48 h. They were then immersed in phosphate buffer 0.1 M (12 h) and cryoprotected with 30% sucrose in PB, immersed in OCT compound (Tissue Tek, Torrance, CA), and frozen with liquid nitrogen-cooled isopentane. Parallel series of transverse sections (18/20 *μ*m thick) were cut on cryostat and mounted on Superfrost Plus (Menzel Gläser, Madison, WI) slides. For immunohistochemical analysis, parallel sections were pretreated with H_2_O_2_, in phosphate-buffered saline at 37°C for 15 minutes to eliminate endogenous peroxidase, rinsed twice in 0.05 M Trizma buffered saline containing 0.1% Tween–20 at pH 7.4 (TBS–T) for 10 minutes each, pretreated with blocking Avidin/Biotin kit, and then incubated overnight with the primary antibodies (monoclonal anti-*β*-amyloid, antiglial fibrillary acidic protein, anti-CD45RA, anti-CD3, antisynaptophysin, and polyclonal antineurofibrillary tangle-like structures; Table 1). The procedure provided by the mouse-on-mouse peroxidase immunodetection system (M.O.M. Kit; Vector) was used to eliminate any nonspecific binding of anti-mouse secondary antibodies with the endogenous mouse immunoglobulins in the tissue, according to the manufacturer's instructions. The sections were successively rinsed in TBS–T, incubated in goat IgG anti-rabbit (Dako) or goat IgG anti-mouse (Dako), depending on the primary antibody, for 1 hour, rinsed in TBS-T, and then incubated for 30 minutes in ABC kit system (Vectastain; Vector). Peroxidase reaction was performed with 3,3-diaminobenzidine as chromogen and hydrogen peroxide as oxidant. In several adjacent sections, negative controls performed by omitting the primary, secondary, or tertiary antibodies showed no immunostaining. Sections were then dehydrated in graded ethanol and covered with a rapid embedding agent (Eukitt; Fluka). 

### 2.9. Amyloid-*β* Plaque Quantification

The quantification of A*β* plaque was determined in randomly selected microscopic transverse sections per animal group from a total of 7 sections per animal/group and were evaluated using the HIH Image J program by defining region of interest and setting a threshold to discriminate nonspecific staining. All data analysis and measurements were blindly performed by investigators unaware of the treatment protocol. 

### 2.10. Image Preparation

Images were visualized using a microscope (Olympus BX50) and were digitized via a digital camera (DP-10; Olympus). The photographs were then adjusted for brightness and contrast with Corel Photo-Paint (Corel, Ottawa, Canada), and plates were composed with Corel Draw. 

## 3. Results

### 3.1. Characterization and Quantification of *β*-Amyloid-Plaques in APP/PS1 Transgenic Mice

**Figure 2:**
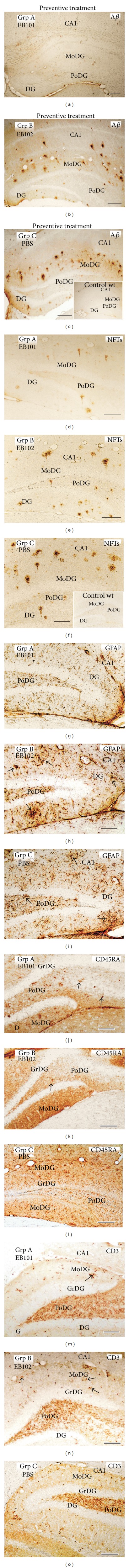
EB101 vaccine prevents development of AD hallmarks in the brains of PS1/APP transgenic mice. Comparative photomicrographs of hippocampal brain regions at the end of preventive treatment (phase I) showing the effects on the AD-like hallmark reduction through immunoreactivity against A*β* ((a)–(c)), NTFs ((d)–(f)), GFAP ((g)–(i)), CD45RA ((j)–(l)), and CD3 ((m)–(o)).

**Figure 3:**
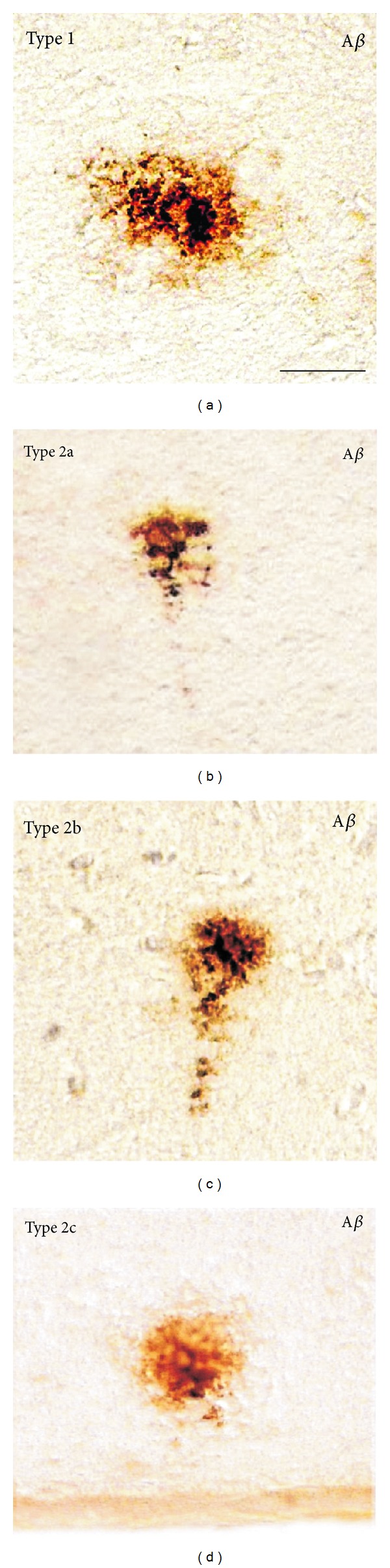
Different types of *β*-amyloid plaques in APP/PS1 mouse brains. (a)–(d): Different types of *β*-amyloid plaques observed in APP/PS1 mice based on the morphological classification: type 1 plaque (a), devoid of a central dense core and displaying an irregular shape; type 2a plaques (b) show a compact shape and exhibit a central core surrounded by fibrillar material; Plaque 2b (c), characterized by fibrillar material radiating from the central dense core; type 2c plaques (d) show a strong *β*-amyloid positivity without any surrounding fibrillar material.

**Figure 4:**
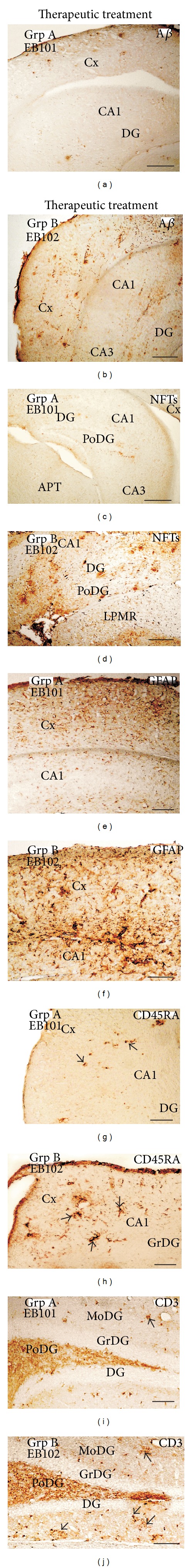
Clearing effect of established AD hallmarks by EB101 vaccine in the brains of PS1/APP transgenic mice. Transverse brain sections of 21-month-old mice of the treatment period (phase II), showing almost complete reduction of AD hallmarks after EB101 vaccine immunization compared with EB102-immunized mice and PBS control groups.

**Figure 5:**
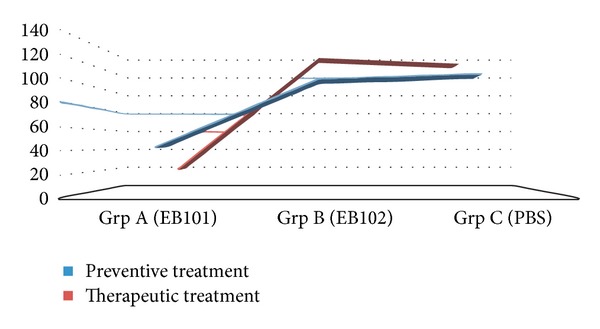
A*β* plaque quantification. The density quantification diagram of A*β* burden observed in the hippocampal and cortical regions of APP/PS1 mice in the three treatment groups at the end of preventive and therapeutic periods. The mean of the A*β* burden (plaques/section) is reduced in group A when compared with groups B and C during both treatment periods and decreased in density from preventive to therapeutic in group A, markedly different to that observed in groups B and C.

In our first experiment (phase I), we investigated the possible preventive effects of the EB101 vaccine in APP/PS1 double-transgenic mice (7 weeks of age) before the appearance of and during the early onset of the Alzheimer-like neuropathology. Brain sections obtained from APP/PS1 transgenic mice after experimental phase I showed many AD-like pathological features (Figures 2(a)–2(o)), including extensive deposition of extracellular *β*-amyloid plaques, astrocytosis, and cerebral inflammation. The histological analysis of control APP/PS1 transgenic mice (groups B and C) revealed four different types of *β*-amyloid plaques (Figures 2–4) based on the morphological characterization of Bussière and colleagues [40]: type 1 plaques were devoid of a central dense core and displayed an irregular shape; type 2a plaques exhibited a central core surrounded by fibrillar material; plaque 2b was characterized by fibrillar material radiating from the central dense core; type 2c plaques, also called “burned-out” plaques by analogy with human classifications, were strongly *β*-amyloid-positive without any surrounding fibrillar material (Figure 3). These *β*-amyloid plaques were mainly present in the hippocampus (Figures 2(b) and 2(c)), notably dense in dentate gyrus (granular layer), followed by neocortical regions such as retrosplenial areas, ectorhinal and piriform cortex layers. After passive immunization, the areas in both the hippocampus (Figure 2(a)) and cerebral cortex of group A transgenic mice (EB101) resulted in scarce density of amyloid-*β* deposits (±42 plaques/section) and differed significantly from those in group B (EB102, ±103 plaques/section), which shows greater A*β* burden densities (Figure 2(b)). Group C mice showed variable density of A*β* plaques (Figure 2(c), ±109 plaques/section), compared to A and B. Wild-type mice showed no A*β* deposits in any brain region (see squared area in Figure 2 (Grp C)). Taken the group C as control in the *β*-amyloid-positive plaque density quantification, the group A showed a preventive effect of 61.3% in the burden plaque reduction while the group B showed only 5.6% (Figure 5).

Figures 2(a)–2(c) show transverse brain sections of 11-month-old mice, at the end of preventive treatment, showing almost complete prevention of A*β* load development after EB101 vaccine immunization (a) compared with EB102-immunized mice (b) and PBS (c). Wild-type control mice showed no brain A*β* plaques (squared area in Figure 2(c)). (d)–(f) show transverse sections of the hippocampal and cortical regions showing a few sparse immunoreactive NFTs in group A (d) with reduced immunoreactivity, which contrasts sharply with the numerous immunoreactive NFTs in the corresponding mouse brain sections of groups B (E; EB102) and C (F; PBS). Note the abundant density of NFTs in these two control groups (E, F), although the hippocampal regions of group B (e) show slightly less density of immunoreactive NFTs compared with those of group C (f). In (g)–(i), transverse panoramic sections of the dentate gyrus show scarce dystrophic astrocytes in group A (g), which contrasts with densely dystrophic reactive astrocytes observed in the corresponding mouse brain sections of groups B (H; EB102) and C (I; PBS). Note the abundant density of dystrophic reactive astrocyte clusters in groups B and C, showing a typical pro-inflammatory pathological pattern. (j)–(l) show transverse brain sections of APP/PS1 mice at the end of preventive period, where an abundant density of immunoreactive B cells was observed in EB101-immunized mice (j) when compared with mice injected with EB102 (k) and controls (l). Note the almost absence of immunoreactive B cells in the EB102-treated mouse brain sections (k), whereas the hippocampal sections of the EB101-immunized mice (j) show a moderate immunoreactivity in response to the neuropathological pro-inflammation process. In (m)–(o), transverse hippocampal sections, show a significant reduction in T-cell aggregation in group A (m) compared with mice immunized with EB102 (n) and PBS (o), where numerous clusters of immunoreactive B cells were mainly observed in the outer layers of the dentate gyrus, suggesting an ongoing neuropathological process.

In the second experiment (phase II), we investigated the possible attenuation effects of the EB101 vaccine in APP/PS1 transgenic mice (35 weeks of age) after the appearance of the Alzheimer-like neuropathology by performing the same methodological analysis as described for the previous phase I. The histological analysis of APP/PS1 transgenic mice after the therapeutic immunization period showed a notable increase in the pathological features compared to that observed in the preventive period (Figures 4(a)–4(j)), mainly observed in experimental transgenic mice of groups B and C. Brain sections of the main affected areas related to AD-like pathologies of transgenic mice immunized with EB101 (group A) showed a significant reduction (84.6%) in, and almost a complete absence of, A*β* deposits (Figure 4(a), ±21 plaques/section), markedly different from the A*β* burden densities observed in transgenic mice of groups B (EB102; Figure 4(b)) and C (PBS), (Figure 5). The few *β*-amyloid plaques observed in mice of group A presented a tiny central core surrounded by scarce fibrillar material (type 2a), and they were mainly located at the external cortical layers (Figures 3(a) and 4(a)). In the brain section of the same transverse levels, mice of group B presented an extensive density of *β*-amyloid plaques (Figure 4(b); ±136 plaques/section), showing a scattered distribution throughout the main AD-like affected regions (hippocampal and cortical layers). Transgenic mice of group C showed similar A*β* burden levels (Figure 5) to those observed in transgenic mice of group B. 

The transverse sections of the dentate gyrus, hippocampal subregion CA1, and cortical areas show scarce and sparse A*β* plaques with weak immunoreactivity in the dentate gyrus of group A (a), contrasting notably with the numerous A*β* immunoreactive plaques in the corresponding mouse brain sections from group B (b). Note the abundant density of A*β* immunoreactive plaques (b), as well as their extensive size and stronger immunolabeling when compared with group A brain sections Figures 4(a) and 4(b). In (c)-(d), transverse hippocampal sections show a few small sparse immunoreactive NTFs in group A (c) that markedly contrast with the numerous strongly immunoreactive NFTs observed in the corresponding mouse brain sections from group B (D; EB102). In (e)-(f), transverse mouse brain sections of the treatment period show almost complete reduction of astrocytosis after EB101 vaccine immunization (e) compared with EB102-immunized mice (f). Note that transverse sections of the retrosplenial cortex/hippocampal subregion CA1 show areas devoid of or with very few visible reactive astrocyte clusters (e), contrasting with the numerous immunoreactive GFAP clusters observed in the corresponding mouse brain sections from group B (f). (g)-(h) show comparative photomicrographs of hippocampal and cortical brain regions at treatment period, showing a moderate to scarce density of immunoreactive B cells at the dentate gyrus, restrosplenial cortex, and hippocampal subregion CA1 of EB101-immunized mice (g), contrasting markedly with the massive immunoreactive density of B cells observed in EB102-immunized mice (h). Note the extensive response to the neuropathological inflammation process in brain sections of group B mice (h). (i)-(j) Transverse hippocampal sections of EB101-treated mice show scarce density of immunoreactive T cells in the dentate gyrus (i), contrasting with a significant increase in immunoreactive T cells observed in the same brain area of EB102-immunized mice (j). For abbreviations, see list. Scale bar: 100 *μ*m.

### 3.2. Characterization of Neurofibrillary Tangle-Like Structures in APP/PS1 Transgenic Mice

Using antibody against neurofibrillary tangle-like structures (NFTs), we observed stained tangles in all immunized groups (Figures 2(d)–2(f)) at the end of phase I, although the density of these AD-like hallmarks was substantially higher in mice brains of group B (Figure 2(e)) and C (Figure 2(f)). The immunopositive structures observed, formed by aggregations of hyperphosphorylated tau protein along the neuronal helical filaments, showed a plaque-like immunoreactive core with an apical variable dendrite extension, often having a flame-shape appearance. The cytological results obtained in the APP/PS1 transgenic mice of group C after the preventive immunization period (Figure 2(f)) were similar to those observed in mice in group B (Figure 2(e)), where an extensive density of NFTs occupied all AD-like affected regions (hippocampal regions, retrosplenial areas, and ectorhinal and piriform cortex). The same affected brain regions of APP/PS1 transgenic mice immunized with EB101 (group A) were almost devoid of NFTs (Figure 2(d)), only a scattering being observed in the dentate gyrus and entorhinal cortex layers. The stained intensity of NFTs observed in control groups, as well as their density and burden area, contrasts markedly with the scarcity and almost absence of these neuropathological features in EB101-treated transgenic mice. Wild-type mice presented no plaques in any brain region (see squared area in Figure 2(f)). The immunocytological analysis of APP/PS1 transgenic mice after the treatment immunization period of phase II (Figures 4(c) and 4(d)) showed that control transgenic mice presented a similar distribution pattern of NFTs observed in control mouse brain sections of the preventive period (Figures 2(d)–2(f)), although the density and the degree of the immunoreactive staining level were notably increased. This particular neuropathological feature was scarce in transgenic mice immunized with EB101 at this experimental period (Figure 4(c)), similar to that observed in the preventive period, where both adjacent sections were almost devoid of stained NFTs.

### 3.3. Astrocytosis and Reactive Glial Response in APP/PS1 Mice

The effects of the preventive and treatment immunization on the inflammation process have been investigated by the determining of the expression of astrocyte markers (GFAP) in transgenic mouse brains. After the preventive immunization period, activated and/or reactive glia were observed surrounding neuronal plaques (*β*-amyloid plaques and NFT-like structures), showing a conspicuous morphologic organization in the cortical and hippocampal brain regions of control transgenic mouse brains (Figures 2(g)–2(i)). EB101-immunized mouse brains from group A (Figure 2(g)) were almost devoid of reactive gliosis, whereas the APP/PS1 transgenic mice of groups B (Figure 2(h)) and C (Figure 2(i)), showed a substantially high density of GFAP immunoreactivity in the same brain areas with astrogliosis-like morphology (aggregates of astrocytes replacing nearby death neurons). The wild-type mouse group showed a normal distribution of astrocytes throughout the brain regions, where no astrocytosis and/or gliosis were observed (data not shown). After the treatment immunization period, immunocytochemical analysis of APP/PS1 transgenic mice from group A (Figure 4(e)) showed a glial distribution pattern similar to that observed in wild-type mice, where no reactive glia were observed, except conspicuously in a few cortical areas (Figure 4(e)). The overall distribution pattern of GFAP immunopositive astrocytes showed absence of astrocytosis as well as gliosis in the mouse group treated with EB101, in contrast with that observed in control groups B (Figure 4(f)) and C. 

### 3.4. Synaptic Distribution in APP/PS1 Transgenic Mouse Brains

To study whether synaptic density alteration occurs during both experimental periods of immunization, we analyzed the cytoarchitecture and neuronal distribution of the presynaptic marker (synaptophysin) in all mouse brains. In preventive phase I, we observed no significant differences or density variation among the mouse brains of the three groups analyzed. In phase II (therapeutic treatment), transgenic mice from groups B and C showed a slight decrease in synaptophysin density, mainly in the hippocampal regions, although we found no significant difference (data not shown). 

### 3.5. Reactive Immune System in APP/PS1 Transgenic Mouse Brains

To assess the effect of immunization treatment on brain immune reactivity, we analyzed the distribution of the T-cell (CD3) and B-cell (CD45RA) surface markers in the transverse section of all mouse brains. Photomicrographs from brain sections from phase I show some immunoreactive B cells in mice from group A (EB101), especially at the hippocampal regions (Figure 2(j)), this leukocyte surface marker being of a lower density in the mouse brains of group B (EB102; Figure 2(k)) and C (PBS; Figure 2(l)). Very few or no such immune B cells were observed in any of the wild-type mouse brain sections studied. Similar immunoreactive B-cell density was observed in mouse brains of group A (Figure 4(g)), at phase II. However, EB102- (group B; Figure 4(h)) and PBS- (group C) treated mice showed a pronounced increase in CD45RA surface marker density in the brain sections analyzed, mainly at the cortical brain areas affected by fibrillar amyloid-containing plaques. Immunoreactive T cells were found in brain sections of both treated groups A (Figure 2(m)) and B (Figure 2(n)) at phase I, forming conspicuous aggregates in the hippocampal and cortical regions, although these immune T-cell aggregates were slightly lower in the mouse brains from group A (EB101; Figure 2(m)). No such immune cell aggregates were observed in mice of group C (Figure 2(o)). After therapeutic treatment (phase II), T-cell density in the EB101-treated group (Figure 4(i)) showed a similar density to the preventive phase I. However, groups B (Figure 4(j)) and C showed a pronounced increase in CD3 surface markers in the cortical brain areas with dense fibrillar A*β*-containing plaques. These mouse groups showed numerous T-cell clusters in hippocampus and rostral neocortex, consisting of CD3-immunoreactive cells in areas rich in amyloid plaques. 

Moreover, A*β* plaque quantification study showed the A*β* burden observed in the hippocampal and cortical regions of APP/PS1 mice in the three treatment groups at the end of preventive and therapeutic periods (Figure 5). The mean of the A*β* burden (plaques/section) is reduced in group A when compared with groups B and C during both treatment periods and decreased in density from preventive to therapeutic in group A, markedly different to that observed in groups B and C (Figure 5).

## 4. Discussion

Our study confirms reported data demonstrating an early appearance of age-related amyloid plaque formation in APPswe/PS1dE9 mice. A*β* immunostaining showed that there is a variety of different types of *β*-amyloid plaques at early stages, although the majority of plaques were tiny and compact (phase I); and both compact and diffuse plaques were observed at later stages, as shown in the therapeutic protocol (phase II). In other APP-mutated models, the appearance of A*β* plaques changes from 9-10 weeks of age in TgCRND8 mice harboring the human APP gene with the Indiana and Swedish mutations [45] to 6 months of age in APP23 mice expressing human APP with the Swedish double mutation (670/671 KM/NL) [46] or between 9 and 12 months of age in Tg2576 mice carrying a transgene coding for the 695 amino-acid isoform of human APP derived from a large Swedish family [12, 47]. This difference in the time-course pattern of A*β* deposition among different animal models may be caused by the difference in transgenes and mouse genetic backgrounds [48]. At the end of the preventive protocol, non-EB101-treated mice of 11 months of age showed four different types of A*β* plaques, similar to the morphology of thioflavin-S-positive amyloid plaques described by Bussière and colleagues [40]. However, we found that hippocampal and cortical brain areas of transgenic mice of group A (EB101) resulted in scarce density of amyloid-*β* deposits after both preventive and therapeutic immunizations. 

The accumulation of A*β* deposits leads to the activation of several pathways in cells closely associated with the deposits. In order to better understand AD neuropathology, several mechanisms of A*β* clearance via immunotherapy have been proposed in the past decades by using transgenic APP mouse models [41]. Based on previous results, A*β* antibodies may prevent A*β* aggregation and/or dissolve preformed A*β* aggregates [49, 50]. This can be achieved by binding the Fc portion of the antibodies to the Fc receptor on microglia, inducing phagocytosis of A*β* [51]. However, some studies have demonstrated that Fc-mediated phagocytosis is not required for immunotherapy-induced A*β* clearance, as Fab fragments of A*β* antibodies (missing the Fc region) may clear A*β* when applied to the surface of APP transgenic mouse brain [52] or when A*β* vaccination is administrated to APP transgenic mice lacking the FcR-lowered cerebral A*β* [53]. Another mechanism that may not be mutually exclusive suggested that certain antibodies bind A*β* oligomers, thereby neutralizing the toxic effects of this A*β* species on synapses [54]. In the present study, we observed that there is a direct relationship between the increase in the amount of endogenous A*β* antibodies and the clearance of A*β* plaques, suggesting that a combination of the previous proposed mechanisms may play an important role in the notable A*β* clearance induced by immunotherapy. 

There is evidence supporting the important role of inflammation in the pathogenesis of AD. Activated microglia and astrocytes are found in AD patients and transgenic mouse models, located in close proximity to senile plaques [55, 56]. It is known that activated microglia, astrocytes, and amyloid deposition increase with age in the hippocampal and cortical areas in AD mouse models [57, 58]. In the APP/PS1 mouse model, we observed clustered astrocytes in hippocampus and neocortex at 11 months of age. Our study showed that clusters of activated astrocytes were scarce and scattered in EB101-treated mice, in close association with amyloid deposits. Consistently, this association pattern, with a large number of GFAP-positive astrocytes surrounded amyloid plaques, has also been reported in TgCRND8 mice harboring the human APP gene with the Indiana and Swedish mutations [45] and in APPswe/PS1dE9 mouse brains [59]. Results in EB101-treated mice seemed to confirm the beneficial process effect of A*β* clearance by activated microglia and reactive astrocytes [48, 60]. The activation of astrocytes in both preventive and therapeutic protocols observed by immunohistochemical examination may result via the effect of cytokines released from glial cells in contact with aberrant neurons containing A*β* oligomers, as suggested by Duyckaerts and colleagues [61]. In APP mice, both diffuse and fibrillar deposits were associated with activated astrocytes and microglia. However, in human AD brains, approximately half of the diffuse (nonfibrillar) deposits are not associated with activated microglia, and a large percentage of AD brains show single microglia colocalized with diffuse plaques [62]. In contrast, nearly all compact deposits have single or multiple activated microglia embedded in their core [63, 64]. Our results, obtained from brain sections of transgenic mice under therapeutic protocols, show plaque morphologies more closely representing compact, dense cores, which may explain why more plaques are associated with glial reactivity. In preaged transgenic mice, we observed that the association of reactive astrocytes with deposits in some regions of the cortex is diminished. Whether the early EB101-treated mice with a low population of plaques associated with reactive astrocytes represent the type of burned-out plaques occasionally seen in some late stage human AD cases [65] remains to be studied. Many studies argued that one of the effects of anti-A*β* immunotherapy is a shift in the phenotypic state of the activated microglia, leading to a profound inflammatory state which could be neurodestructive [30]. The polar extremes of these activation states have been referred to as Th1 and Th2 adaptive responses [66]. Here, we showed that EB101 mice exhibit increased levels of proliferative immunoreactive T cells, as well as reduced levels of A*β*-reactive B cells and GFAP aggregates. Together with the high levels of IgG antibody production, this may indicate that EB101 does not induce autoimmune encephalitis or other significant signs of inflammation, as measured by immunological markers [67] or by marked astrocytosis [68] in EB101-immunized mice, in clear contrast to that observed in the EB102 and PBS mouse groups. Based on these results, the activation state of inflammation is reduced and localized in neurodegenerative plaques, representing an optimal state for the preventive and therapeutic treatments intended to ameliorate neurodegeneration.

Many active A*β* vaccines now target the amino terminus of A*β* to generate a strong humoral response and avoid an A*β*-specific T-cell response, thought to account for the adverse effects reported in the AN1792 trial. It appears that A*β* immunotherapy, like other A*β*-lowering therapies, may be effective when given before or in the early stages of cognitive decline, prior to massive neuritic dystrophy and neuronal loss [69]. However, the present vaccine reduced neurodegenerative hallmarks in APP/PS1 transgenic mice in both preventive and therapeutic protocols. The immunohistochemical characterization of AD hallmarks observed in transgenic mice in this study shows that the EB101 vaccine may not only halt AD-related pathology but also may actually attenuate it, leading to a robust immunological response and an anti-inflammatory effect as well. In summary, the present results suggest that the EB101 immunization protocols used in this study notably reduce the neuronal pathology associated with AD in APP/PS1 mouse models and offer a novel strategy of effective immunotherapy and prevention in human AD.

## 5. Conclusions

In conclusion, we have studied the immunocytochemical characterization of AD hallmarks to assess the new therapeutic potential of the EB101 vaccine in APP/PS1 transgenic mice. The main findings of current study are (i) beta-amyloid plaques, the main AD-like hallmarks in the hippocampal and cortical regions, were strongly diminished in phase I, where APP/PS1 transgenic mice immunized with EB101 showed few plaques with a tiny and compact morphology, whereas in phase II, compact and diffuse plaques were reduced by this immunization; (ii) a significant reduction in neurofibrillary tangle-like structures, activated astrocytes, and neuroinflammation response in APP/PS1 transgenic mice treated with the EB101 vaccine was observed by using specific immunocytochemical markers; (iii) EB101-treated mice showed no negative effects on brain cytoarchitecture patterns during the different phases of the experimentation actions. Therefore, the qualitative and quantitative immunocytochemical characterization of the AD hallmarks in APP/PS1 mice treated with EB101 in preventive and therapeutic protocols provides useful information of this mouse model when investigating the neuropathogenic effects of AD. Further expanded studies with different mouse or other animals' models are needed to confirm the effective response when treated with this type of vaccines, as well as adapting this immunotherapy to be effective in human clinical studies.

## Figures and Tables

**Table 1:** Antibodies used for immunohistochemistry.

Antibody	Antigen	Type	Source	Dilut.	Reference
*β*-amyloid	A*β*_1–42_ (mouse)	Mouse monoclonal	Millipore	1 : 1000	[36, 37]
Neurofibrillary tangle-like struc.	NFTs (rabbit)	Rabbit polyclonal	Millipore	1 : 300	[38, 39]
Glial fibrillary acidic protein	GFAP (mouse)	Mouse monoclonal	Sigma	1 : 400	[30, 40]
Synaptophysin	Sph (mouse)	Mouse monoclonal	Sigma	1 : 50	[41]
CD45RA	B cells (mouse)	Mouse monoclonal	Dako	1 : 100	[42, 43]
CD3	T cells (rabbit)	Rabbit polyclonal	Dako	1 : 100	[43, 44]
